# Supplementary material for: Integrative Metabolome and Transcriptome Analysis Reveals the Regulatory Network of Flavonoid Biosynthesis in Response to MeJA in Camellia vietnamensis Huang
Source: Int J Mol Sci. 2022 Aug 19;23(16):9370. doi: 10.3390/ijms23169370 (PMC9409299; doi:10.3390/ijms23169370)
Supplement: Supplementary file 1 [file ijms-23-09370-s001.zip › Supplementary Figures.pdf]

Attached drawing

A

B

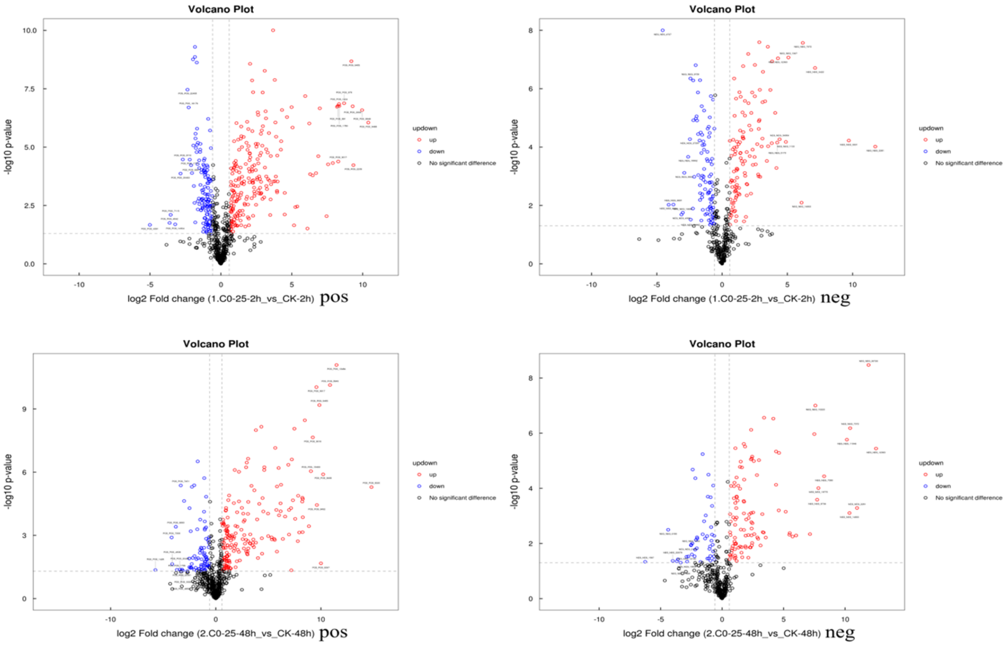

Figure S1. The up-regulation and down-regulation of differential metabolites of *C. vietnamensis* response to MeJA at different time points. TOP 10 metabolites with the most multiples up-down and down-regulation are marked with qualitative names. A: CO-25-2h_vs_CK-2h; B: CO-25-48h_vs_CK-48h.

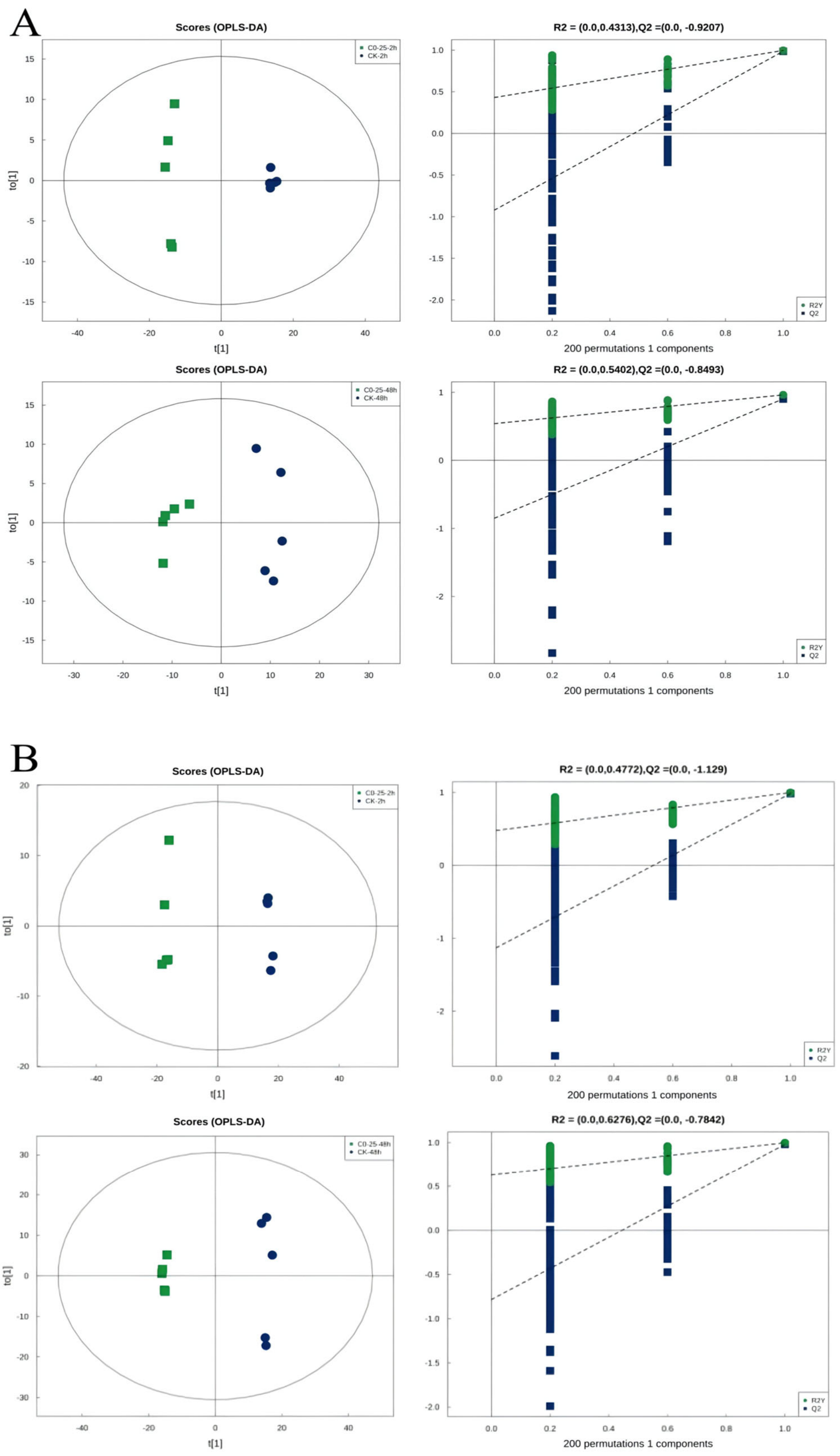

Figure S2. A: The score of the OPLS-DA model and its permutation test in positive ion mode. B: The score of OPLS-DA model and its permutation test in the negative iron mode.

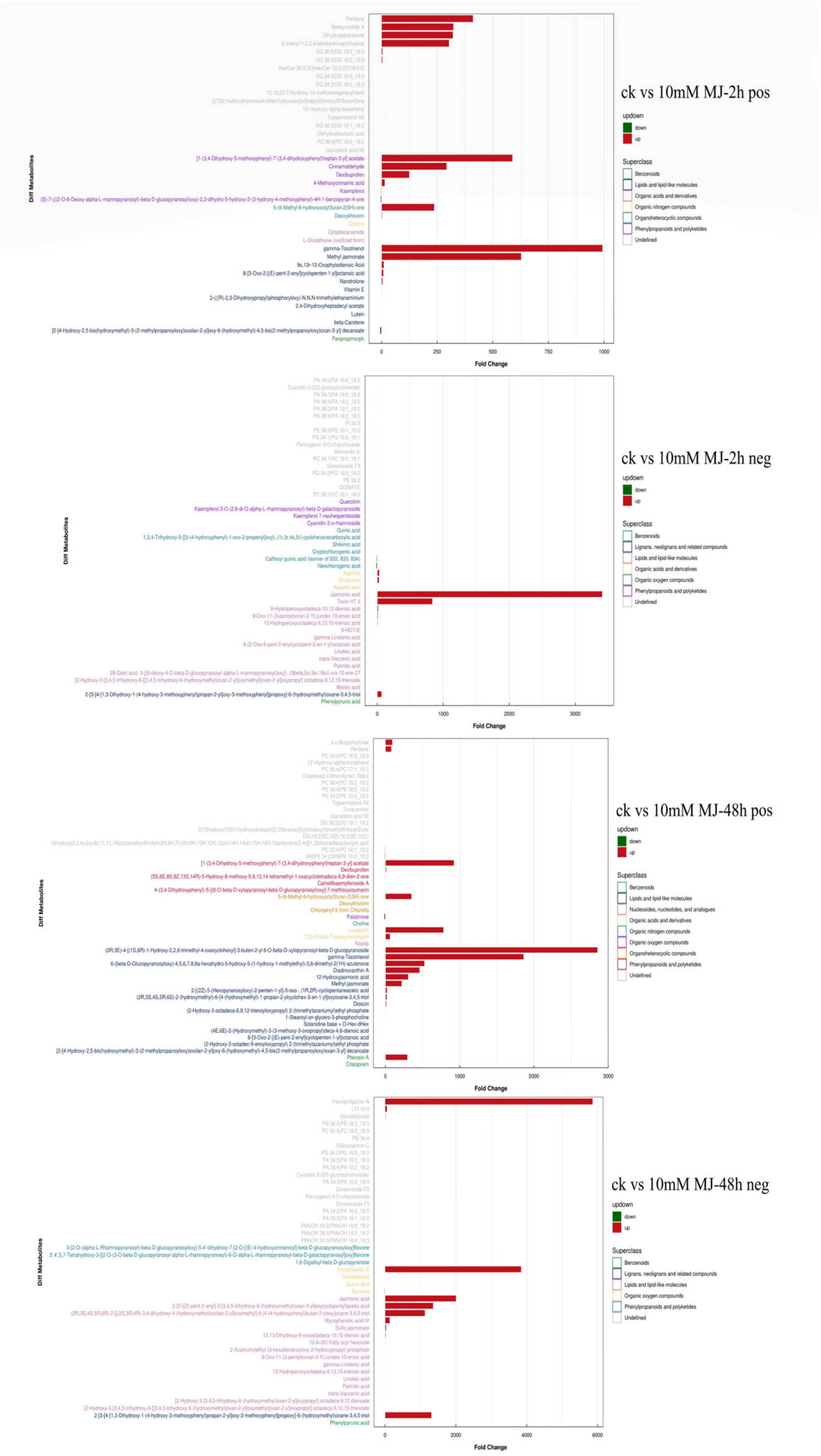

Figure S3. Differentially accumulated metabolites between groups at different times (2 and 48 h) under MeJA treatment.

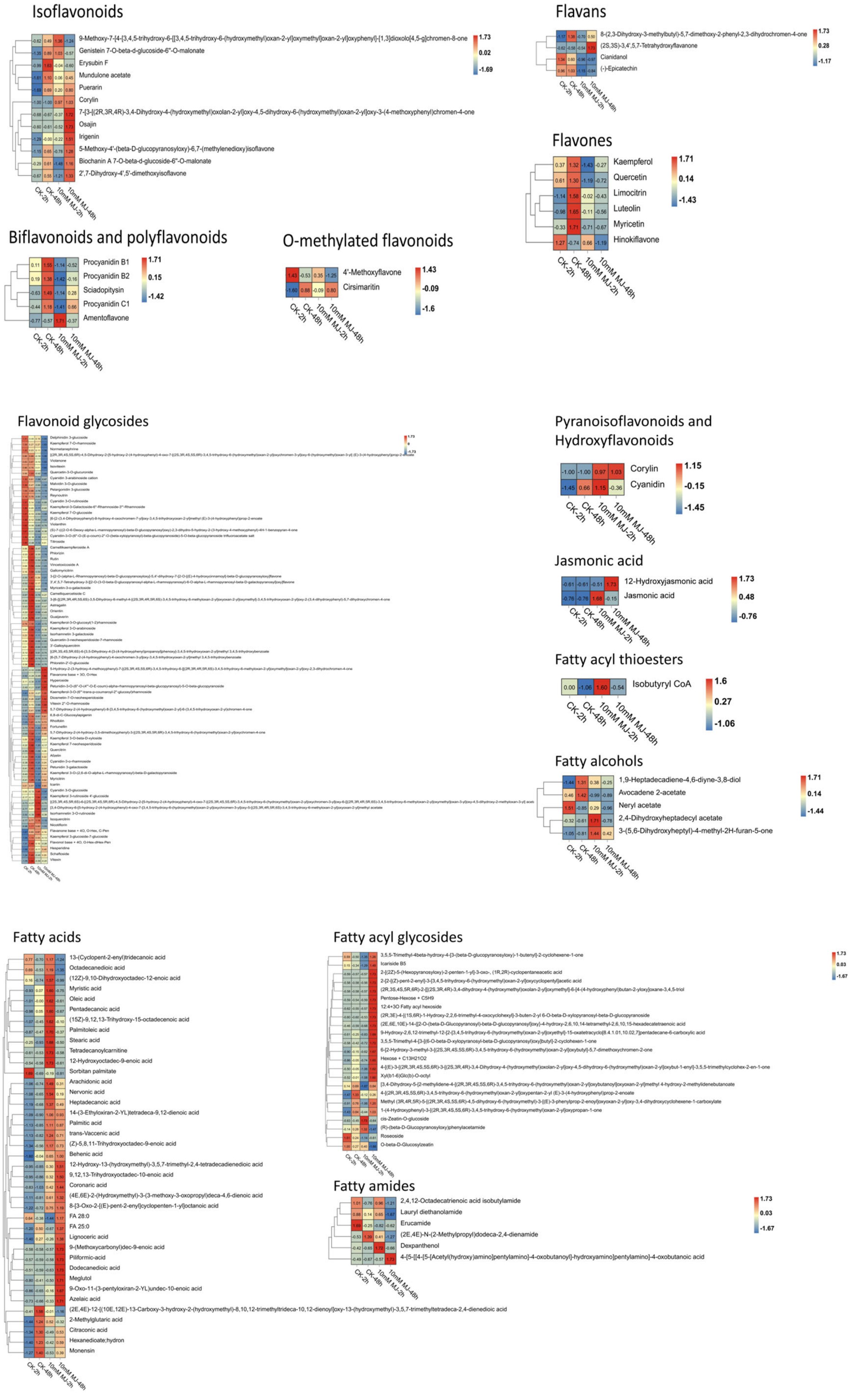
Isoflavonoids
9-Methoxy-7-[4-[3,4,5-trihydroxy-6-[[3,4,5-trihydroxy-6-(hydroxymethyl)oxan-2-yl]oxymethyl]oxan-2-yl]oxyphenyl]-[1,3]dioxolo[4,5-g]chromen-8-one
Genistein 7-O-beta-d-glucoside-6''-O-malonate
Erysubin F
Mundulone acetate
Puerarin
Corylin
Osajin
Irigenin
5-Methoxy-4'-(beta-D-glucopyranosyloxy)-6,7-(methylenedioxy)isoflavone
Biochanin A 7-O-beta-d-glucoside-6''-O-malonate
2',7-Dihydroxy-4',5'-dimethoxyisoflavone
1.73
0.02
-1.69
Flavans
Cianidanol
(-)-Epicatechin
Flavones
Kaempferol
Quercetin
Limocitrin
Luteolin
Myricetin
Hinokiflavone
1.71
0.14
-1.43
Biflavonoids and polyflavonoids
Procyanidin B1
Procyanidin B2
Sciadopitysin
Procyanidin C1
Amentoflavone
1.71
0.15
-1.42
O-methylated flavonoids
4'-Methoxyflavone
Cirsimaritin
1.43
-0.09
-1.6
Flavonoid glycosides
Pyranoisoflavonoids and Hydroxyflavonoids
Corylin
Cyanidin
1.15
-0.15
-1.45
Jasmonic acid
12-Hydroxyjasmonic acid
Jasmonic acid
1.73
0.48
-0.76
Fatty acyl thioesters
Isobutyryl CoA
1.6
0.27
-1.06
Fatty alcohols
1,9-Heptadecadiene-4,6-diyne-3,8-diol
Avocadene 2-acetate
Neryl acetate
2,4-Dihydroxyheptadecyl acetate
3-(5,6-Dihydroxyheptyl)-4-methyl-2H-furan-5-one
1.71
0.14
-1.44
Fatty acids
13-(Cyclopent-2-enyl)tridecanoic acid
Octadecanedioic acid
(12Z)-9,10-Dihydroxyoctadec-12-enoic acid
Myristic acid
Oleic acid
Pentadecanoic acid
(15Z)-9,12,13-Trihydroxy-15-octadecenoic acid
Palmitoleic acid
Stearic acid
Tetradecanoylcarnitine
12-Hydroxyoctadec-9-enoic acid
Sorbitan palmitate
Arachidonic acid
Nervonic acid
Heptadecanoic acid
14-(3-Ethyloxiran-2-YL)tetradeca-9,12-dienoic acid
Palmitic acid
trans-Vaccenic acid
(Z)-5,8,11-Trihydroxyoctadec-9-enoic acid
Behenic acid
9,12,13-Trihydroxyoctadec-10-enoic acid
Coronaric acid
FA 28:0
FA 25:0
Lignoceric acid
9-(Methoxycarbonyl)dec-9-enoic acid
Piliformic-acid
Dodecanedioic acid
Meglutol
9-Oxo-11-(3-pentyloxiran-2-YL)undec-10-enoic acid
Azelaic acid
2-Methylglutaric acid
Citraconic acid
Hexanedioate;hydron
Monensin
Fatty acyl glycosides
Icariside B5
Pentose-Hexose + C5H9
Hexose + C13H21O2
cis-Zeatin-O-glucoside
Roseoside
O-beta-D-Glucosylzeatin
Fatty amides
2,4,12-Octadecatrienoic acid isobutylamide
Lauryl diethanolamide
Erucamide
(2E,4E)-N-(2-Methylpropyl)dodeca-2,4-dienamide
Dexpanthenol
1.73
0.03
-1.67
CK-2h
CK-48h
10mM MJ-2h
10mM MJ-48h

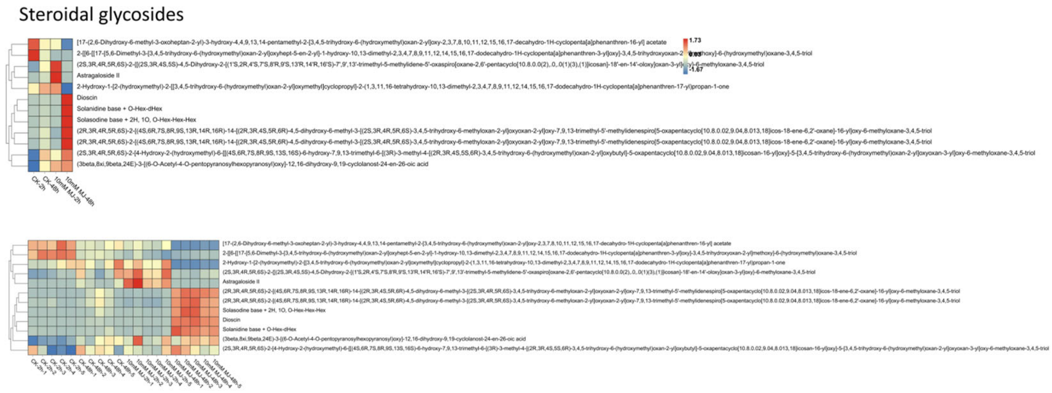

Figure S4. Heatmap of metabolite profile for 121 compounds.

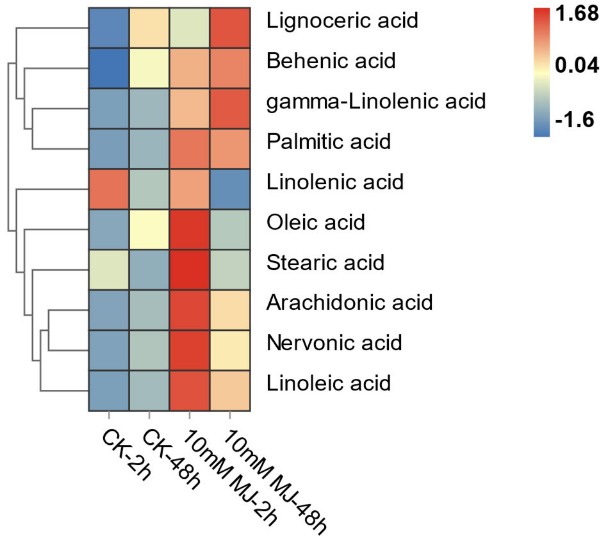

Figure S5. Heat map of fatty acid metabolism pathway metabolites.

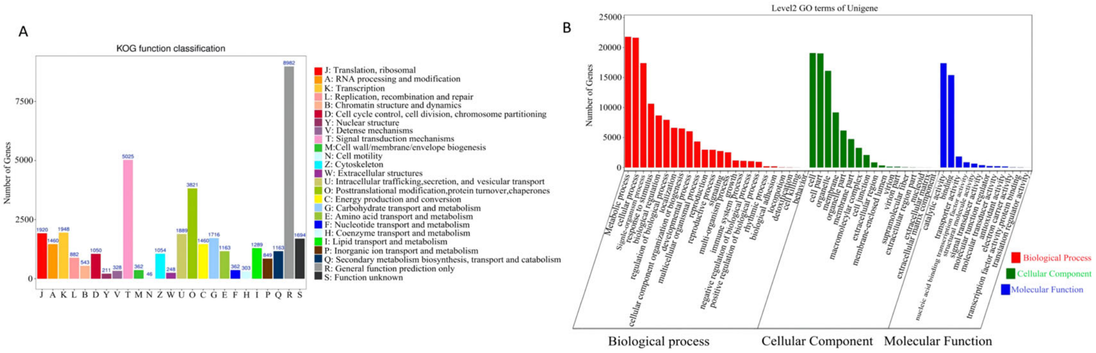

Figure S6. A: KOG functional classification. B: Unigene distribution with GO functional classification.

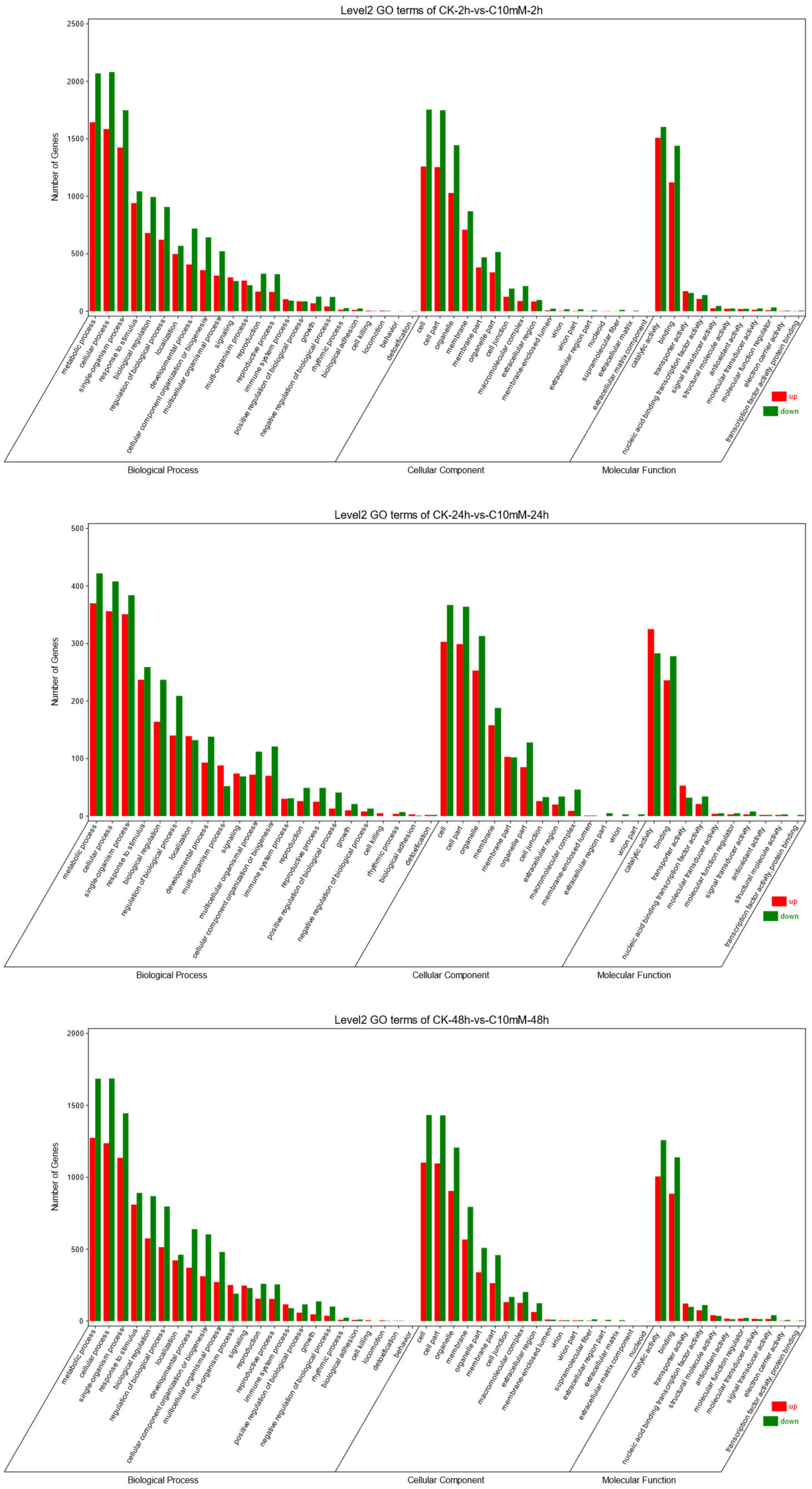

Figure S7. GO annotation classification of differential genes.

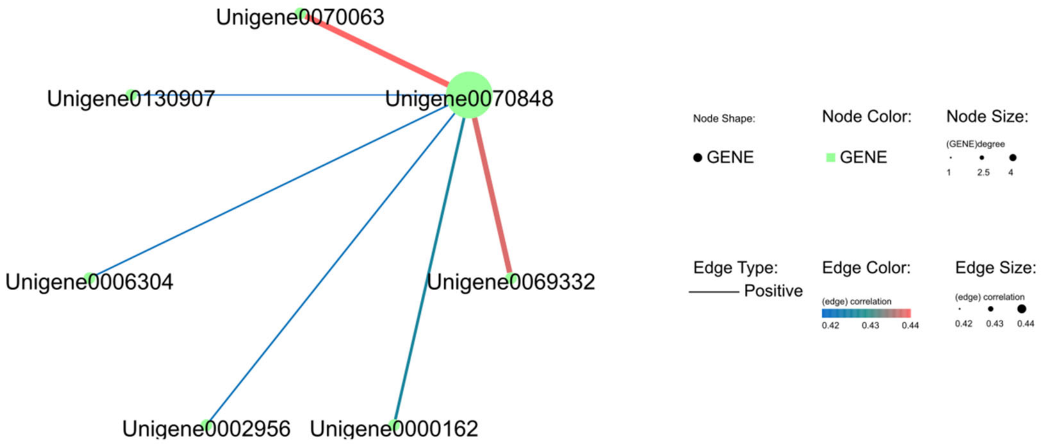

Figure S8. Cytoscape map of Unigene0070848. Each point and line in the figure represents a gene. Each line represents a regulatory relationship between two points. The darker and larger the node color and line, the stronger the connectivity.

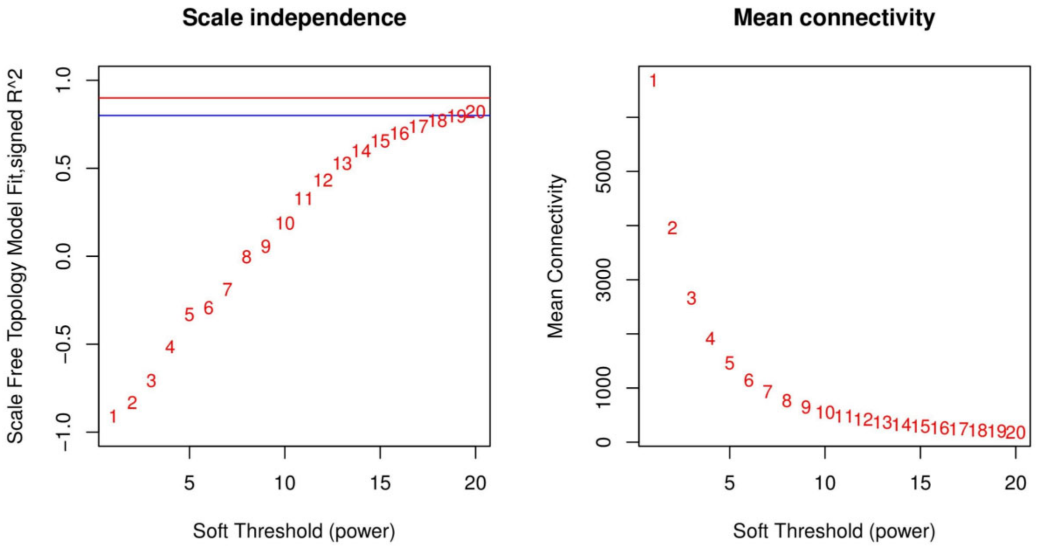

Figure S9. Soft threshold (power) value curve. The calculation of the soft threshold value can reveal the relationship between the correlation coefficient and the average connectivity.

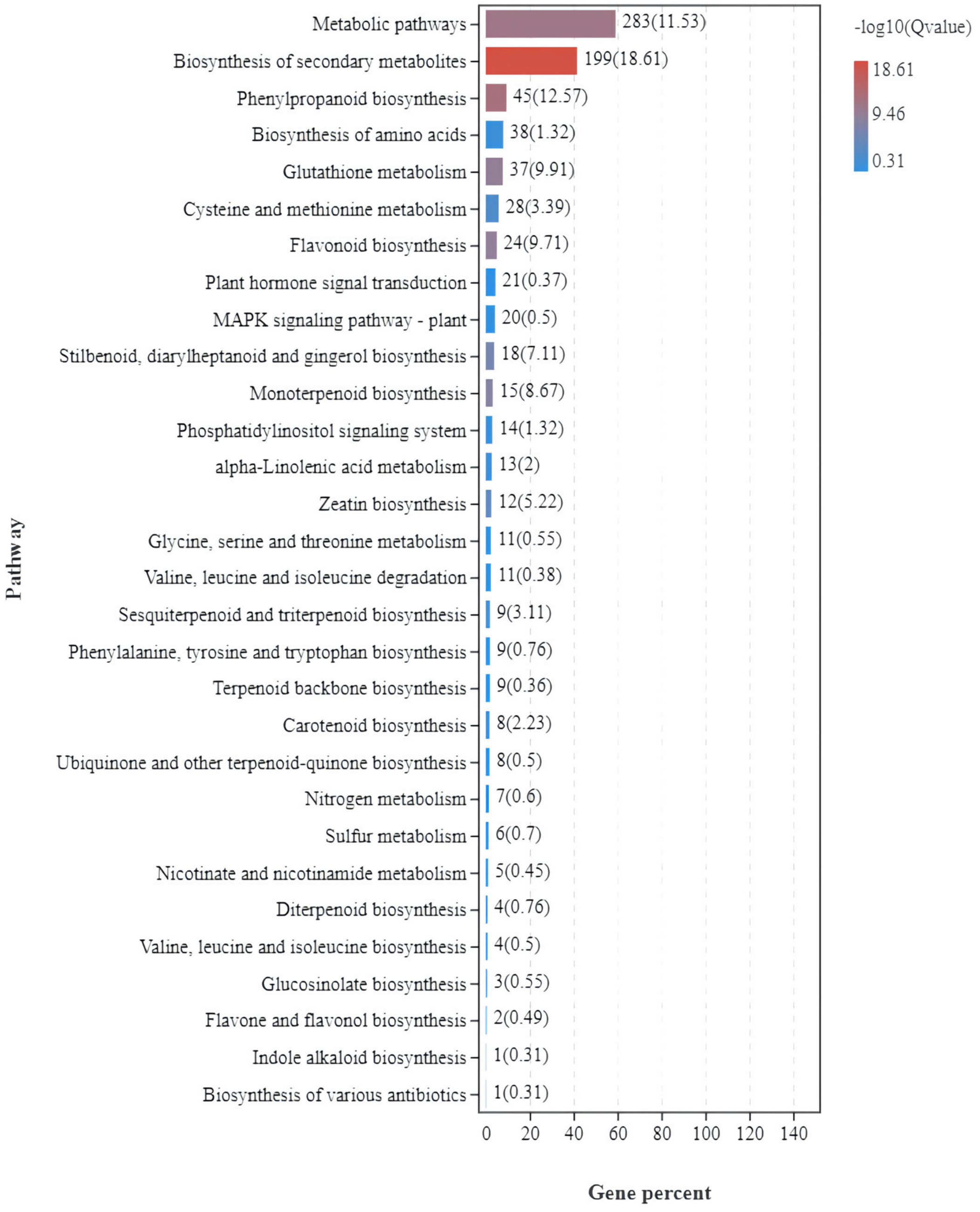

Figure S10. KEGG analysis in blue module.

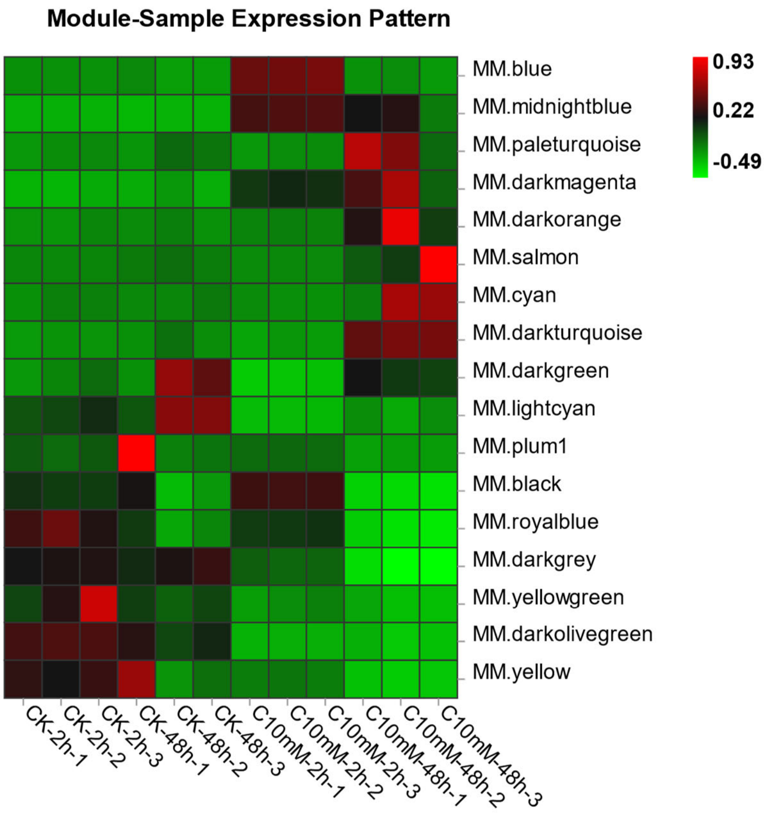

Figure S11. Module-sample expression pattern.

A:

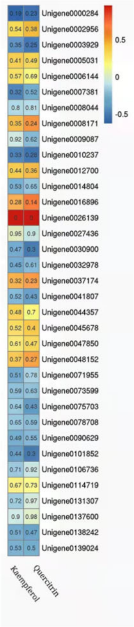

B:

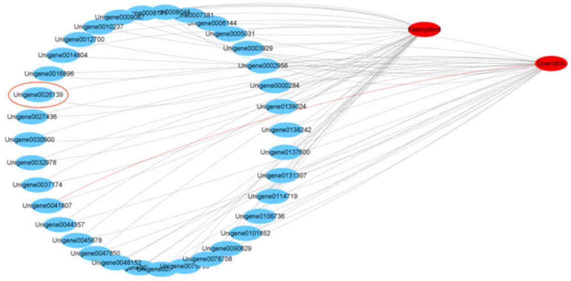

Figure S12. A: Heatmap of correlations between differential flavonoid-related genes and metabolites. Red represents positive correlation and blue represents negative correlation. The darker the color, the stronger the correlation. The number in the box represents the p-value. The smaller the value, the stronger the significance. B: The connection network between the differential flavonoid-related genes and differential flavonoid metabolites. Blue represents genes and red represents metabolites.

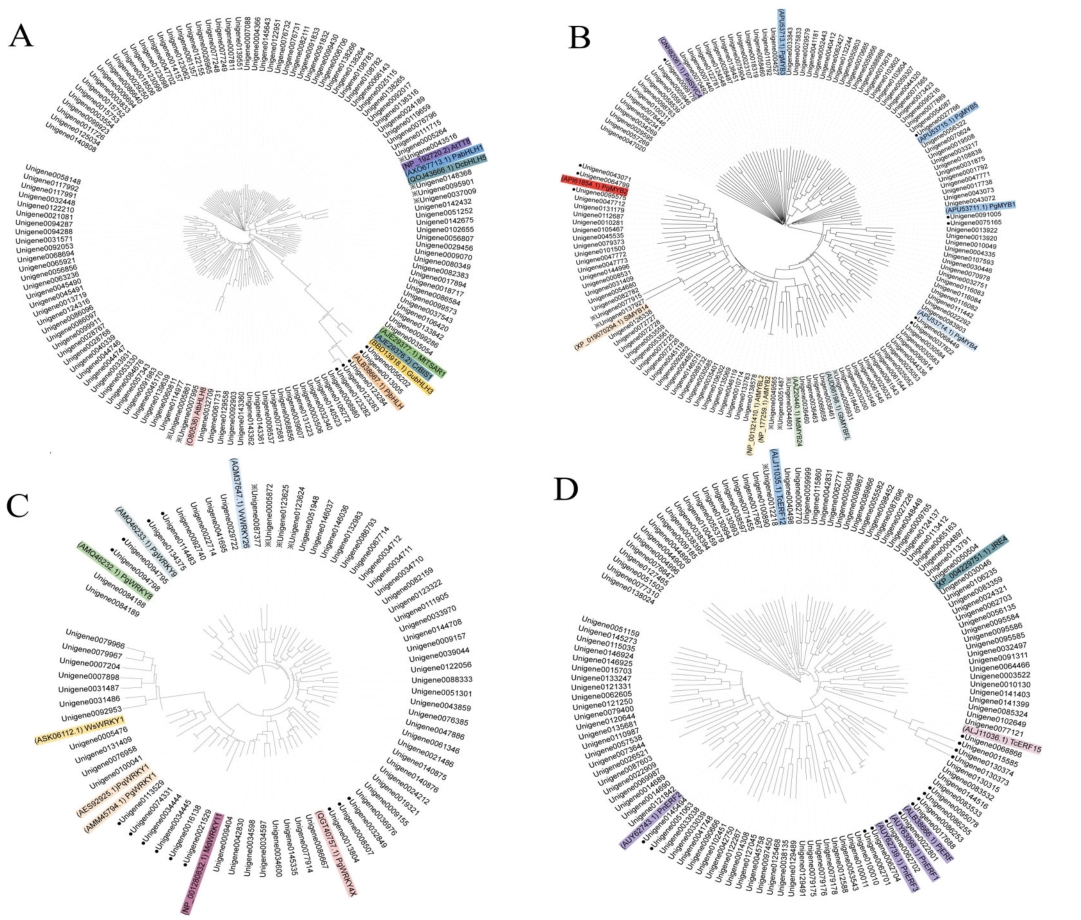

Figure S13. Phylogenetic tree of bHLH, MYB, WRKY, and ERF families. A: bHLH; B: MYB; C: WRKY; D: ERF.
